# Molecular detection of two major gastrointestinal parasite genera in cattle using a novel droplet digital PCR approach

**DOI:** 10.1007/s00436-019-06414-7

**Published:** 2019-08-06

**Authors:** Paulius Baltrušis, Peter Halvarsson, Johan Höglund

**Affiliations:** Department of Biomedical Sciences and Veterinary Public Health, Section for Parasitology, Swedish University of Agricultural Sciences, P.O. Box 7036, Uppsala, Sweden

**Keywords:** Strongyles, ddPCR, *Cooperia*, *Ostertagia*, ITS2

## Abstract

*Cooperia* sp. and *Ostertagia* sp*.* are two cosmopolitan parasitic nematodes often found in mixed gastrointestinal infections in cattle across temperate regions. In light of the recent increase in the emergence of anthelmintic resistance in these and other nematodes derived from cattle around the globe, and their negative impact on animal health and productivity, novel molecular assays need to be put forth in order to facilitate the monitoring of parasite burden in infected herds, using pasture and/or fecal samples. Here, we describe a novel droplet digital PCR platform–based concept for precise identification and quantification of the two most abundant and important parasite genera in grazing western European cattle. By exploiting a single nucleotide difference in the two parasites’ ITS2 sequence regions, we have developed two specific hydrolysis probes labeled with FAM™ or HEX™ fluorophores, which can not only distinguish between the DNA sequences of the two, but also quantify them in mixed DNA samples. A third, newly developed universal probe was also tested along the genus-specific probes to provide a robust and accurate reference. It was evident that the universal probe displayed congruent results to those obtained by the genus-specific probes when used with DNA from both parasites in a single sample. All in all, the results of our assay suggest that this novel protocol could be used to distinguish and quantify cattle parasites belonging to the two most important genera (i.e., *Cooperia* and *Ostertagia*) in a single mixed DNA sample.

**Electronic supplementary material:**

The online version of this article (10.1007/s00436-019-06414-7) contains supplementary material, which is available to authorized users.

## Introduction

Infections with parasitic nematodes in cattle yield poor outcomes for both the animal(s) and the industry. Although gastrointestinal nematode (GIN) infections in ruminants often tend to be mixed, the most commonly encountered genera in European cattle are *Cooperia* and *Ostertagia* (Craig 2018). Whereas *Cooperia* is mostly abundant in younger cattle and does not constitute a grave threat, worms belonging to the genus *Ostertagia* are known to induce severe pathological changes in the abomasum (ostertagiosis), leading to the observation of clinical symptoms such as ill thrift and diarrhea in the affected individuals (Craig 2009).

The monetary damage to the cattle industry is, however, mostly associated with the subclinical effects leading to the decreased milk yield and increased anthelmintic usage, as confirmed by a recent European study (Charlier et al. 2012). Furthermore, reduced response to anthelmintic treatment and/or anthelmintic resistance has been readily identified in both species (Demeler et al. 2009; Höglund et al. 2013; Peña-Espinoza et al. 2016; Ramos et al. 2016; Waghorn et al. 2006). To assume control of GINs in grazing livestock, it is important to monitor both at risk and infected animals, especially keeping track of the levels of infection with the more pathogenic *Ostertagia* worms (Charlier et al. 2010). Although more traditional methods are currently used in both the detection of parasitic nematode species and anthelmintic resistance determination in herds (such as Fecal Egg Count Reduction Test), the scientific community is currently searching for molecular assays to provide quicker, less labor-intensive, and more precise alternatives.

The molecular techniques designed for either of the aforementioned purposes range from the very first few PCR-linked restriction fragment linked polymorphism assays (Gasser et al. 1994) to semi-quantitative, real-time (Höglund et al. 2013), and multiplex tandem (Roeber et al. 2017) PCRs, as well as the recently introduced third-generation droplet digital (dd)PCR techniques (Baltrušis et al. 2018; Elmahalawy et al. 2018b). However, the majority of work and effort into improving the detection of nematode parasites in livestock go into the small ruminant sector, where the problem of drug-resistant worms is the most severe (Wolstenholme et al. 2004).

**Fig. 1:**
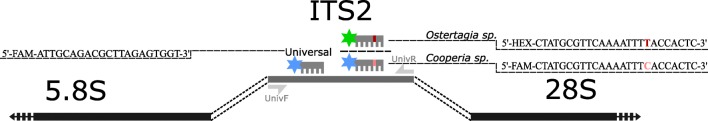
A summary of the assay concept, displaying primers and different, fluorescently -labeled probes for the ITS2 region in genera *Cooperia* and *Ostertagia*. Primers “UnivF” and “UnivR” are displayed as light-gray half-arrows, whereas different probes—“Universal,” “Cooperia,” “Ostertagia” (and their sequences)—are depicted as dark gray rectangles with either FAM™ (blue circle) or HEX™ (green circle) fluorescent dyes attached to them. A single nucleotide difference, found between the consensus sequences of the ITS2 region in both genera of parasites, is highlighted in different shades of red between the two genera specific probes and their sequences

In this paper, we address this knowledge gap between cattle and small ruminants by describing a novel assay concept that relies on the ddPCR platform for the detection and quantification of *Cooperia* and *Ostertagia* genus-specific DNA. The described technique is based on identifying a unique single nucleotide difference in the ITS2 region of genomic DNA between the parasite genera using sequence-specific, fluorescently labeled hydrolysis probes and universal, for that region, primers (Fig. 1). The presented ddPCR assay results constitute an important stepping stone towards the development of an improved diagnostic tool, ultimately used in the monitoring of relative influence of both of these omnipresent parasite genera in grazing cattle herds.

## Materials and methods

### Ethical statement

DNA used in this study was obtained from parasites collected for a previous project. It was conducted with animals and with anthelmintic drugs in compliance with the current laws of the country in which they were performed. Ethical approval was granted by the Committee on Animal Experiments in Gothenburg, Sweden (registration number 187-2014).

### DNA material

DNA was extracted from either frozen larval cultures, isolated from experimentally, mono-specifically infected calves, or adult worms for both *Cooperia oncophora* and *Ostertagia ostertagi*, using NucleoSpin XS Tissue kit (Macherey Nagel, Germany; extraction guidelines are issued by the manufacturer). A single microliter of these solutions was further diluted (if necessary) and used in the ddPCR reactions.

### DNA sequence data

The DNA sequences used for the alignment and comparison of the ITS2 region between the two parasite genera were downloaded from the NCBI database Nucleotide section (https://www.ncbi.nlm.nih.gov/nuccore), using keywords “internal transcribed spacer 2,” “Cooperia,” and “Ostertagia”. It was decided to only keep the sequences belonging to the two most well-known and studied species in European cattle—*Cooperia oncophora* and *Ostertagia ostertagi*. All downloaded sequences for each parasite species were then aligned using CodonCode Aligner (v. 8.0.1), and the subsequent consensus sequences for both parasite species were inspected and compared.

### Amplicon size and sequence confirmation

Primers *UnivR* and *UnivF*, originally developed for a universally conserved region in the ITS2 of the ribosomal RNA gene in sheep strongyles (amplicon size − 109 bp) (Elmahalawy et al. 2018a), were used in concert with the DNA samples, extracted for each parasite genera, in a PCR with the following conditions: a single cycle of 95 degrees for 10 min and 35 cycles of 95 degrees for 15 s, 57 degrees for 30 s, and 72 degrees for 1 min. A single final extension cycle of 72 degrees for 5 min was included at the very end. The amplified products were checked on a 1% agarose gel to confirm their size, then cleaned up using AMPure XP beads (following the guidelines issued by the manufacturer) and sent for Sanger sequencing to validate the composition of amplified sequences.

The obtained trimmed, consensus sequences for the amplicons of each parasite genera were compared using the BLAST algorithm (https://blast.ncbi.nlm.nih.gov/Blast.cgi) with the NCBI “Nucleotide collection” database, and the subsequent results confirmed the origin of the amplified sequences.

### Droplet digital PCR

Droplet digital PCR was run on extracted DNA samples using previously described primers (UnivF and UnivR), as well as three different probes: the Universal-probe (FAM™-ATTGCAGACGCTTAGAGTGGT), Cooperia-specific-probe (FAM™-CTATGCGTTCAAAATTTCACCACTC), and Ostertagia-specific-probe (HEX™-CTATGCGTTCAAAATTTTACCACTC). The detailed process of running the samples using ddPCR technology was described previously (Baltrušis et al. 2018). In short, sample reactions were assembled in 96-well plates (final volume 22 μL), following the guidelines issued by the manufacturer (BioRad). Droplets were generated and dispensed into a new 96-well plate using an automated droplet generator (QX200, BioRad). The new plate was heat sealed and transferred into a thermal cycler (MyCycler™ Thermal Cycler). The PCR conditions were as follows: a single cycle of 95 °C for 10 min, and40 cycles of 94 °C and then 57 °C (optimal annealing temperature; Supplementary figure 1), followed by a single cycle of 98 °C to deactivate the enzyme. After the amplification step, the plate containing the droplets was loaded into the droplet reader (QX200, BioRad) and further analyzed using QuantaSoft (v1.7.4.0917) software, which generates data, including DNA copy measurements and error bars, based on the Poisson distribution (refer to the Applications Guide manual issued by the manufacturer for more information). The output from QuantaSoft was then visualized using the *ggplot2* package (v3.1.0) for *R* software (v3.5.2).

## Results

To validate our newly proposed assay for *Ostertagia* and *Cooperia* genera DNA identification and quantification, we performed a limit of detection (LoD), a fractional abundance (FA) precision, and an absolute quantification-comparison, with both genus-specific and universal (reference) probes, experiments.

**Fig. 2:**
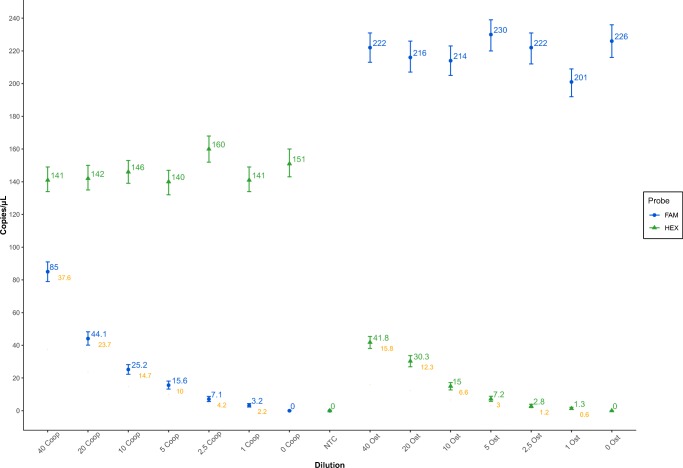
Limit of detection (LoD) assay for the determination of the lowest detectable FA of *Ostertagia* and *Cooperia* DNA in mixed samples. Either *Ostertagia* or *Cooperia* DNA containing samples were diluted (to 40%, 20%, 10%, 5%, 2.5%, and 1% of their initial volume) and mixed with a constant concentration of the other parasite genus DNA. Two negative control samples in each category, containing only a single type of DNA (either *Ostertagia* or *Cooperia*; “0 Coop” and “0 Ost” respectively) in addition to a negative template control (NTC) were also run. Blue filled dots correspond to the copy number of *Cooperia* DNA molecules and green filled triangles to the copy number values for *Ostertagia* DNA. Values displayed in orange represent the FA indices for either *Cooperia* or *Ostertagia* DNA copy number in the mixture

The LoD experiment defined the lowest threshold for the detection of each parasite genus DNA in a mixed sample, containing a gradient DNA dilution of one and a constant DNA copy number presence of the other. We were able to achieve a robust detection of 2.2% of fractional DNA copy number/μL abundance for *Cooperia* ($\frac{\mathrm{Cooperia}\ \mathrm{DNA}\ \mathrm{copy}\ \mathrm{number}/\upmu \mathrm{L}}{\mathrm{Cooperia}+\mathrm{Ostertagia}\ \mathrm{DNA}\ \mathrm{copy}\ \mathrm{number}/\upmu \mathrm{L}}$) and 0.67% for *Ostertagia* ($\frac{\mathrm{Ostertagia}\ \mathrm{DNA}\ \mathrm{copy}\ \mathrm{number}/\upmu \mathrm{L}}{\mathrm{Ostertagia}+\mathrm{Cooperia}\ \mathrm{DNA}\ \mathrm{copy}\ \mathrm{number}/\upmu \mathrm{L}}\Big)$ (Fig. 2). That is to say that 1 molecule of *Ostertagia* DNA can be identified within a mix of 45 DNA molecules, whereas a single *Cooperia* DNA molecule can be picked up in a mix of roughly 149 DNA molecules.

**Fig. 3:**
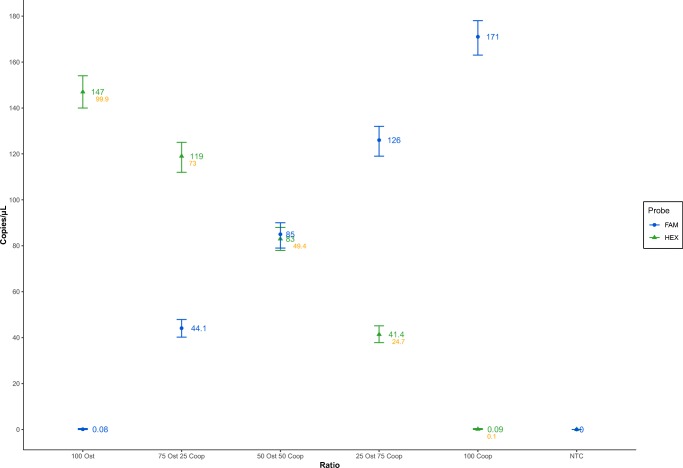
Fractional abundance (FA) precision test. *Ostertagia* and *Cooperia* DNA samples of similar concentrations were mixed in equal volumes at different ratios (1:0, 3:1, 1:1, 1:3, and 0:1) to evaluate the capacity of the technique to determine the FA of each parasite genus DNA at every dilution ratio and produce an anticipated linear dilution pattern. Blue filled dots correspond to the copy number of *Cooperia* DNA in the initial sample, while green filled triangles correspond to the copy number of *Ostertagia* DNA. Values displayed in orange represent the FA index for *Ostertagia* DNA copy number

The FA precision test, wherein both *Ostertagia* and *Cooperia* DNA samples of similar concentrations (DNA copies/μL) were mixed at various ratios (1:0, 3:1, and 1:1), determined the variation in the predicted fractional abundances between the samples to be at most around 2% (in the case of 3:1 dilution for *Ostertagia*:*Cooperia* DNA) (Fig. 3).

**Fig. 4:**
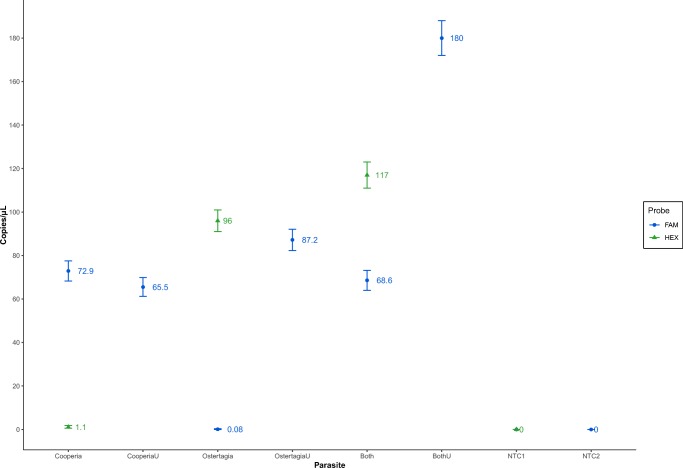
Average number of DNA copies per μl obtained in samples spiked with *Cooperia*, *Ostertagia*, or both parasite genera DNA (v/v 1:1). Each sample was run twice: using genus-specific and universal (e.g., Cooperia and CooperiaU) probes. In addition, two negative template controls (NTC1 and NTC2) were included

The absolute quantification experiments run on DNA derived from both parasites, separately and in combination, using the genus-specific probes and the universal probe produced highly similar results (Fig. 4), apart from a slight increase in the number of copies for *Ostertagia* DNA (from an average 96 ± 5 copies in the “Ostertagia” sample to an average of 117 ± 6 in the “Both” sample, where both parasites are mixed in equal 1:1 volume ratio). In all three of the above-listed experiments, negative template control (NTC) samples did not yield any false positive droplets.

## Discussion

It is generally the case that a healthy balance between parasitic infections with GINs and animal productivity needs to be maintained. This is usually achieved through the use of anthelmintic drugs, which remove the existing worm population and prevent subsequent reinfection of the animals (Vlassoff et al. 2001). While less interest had been expressed in the possibility of anthelmintic resistance development in the parasitic GINs of cattle compared with small ruminants (Coles 2002), the actual numbers of resistant isolates, especially for *Cooperia* spp., appeared to have surged around the globe (Sutherland and Leathwick 2011). To tackle the problem of growing anthelmintic resistance and, thus, the prevalence of cattle GIN parasites, we have developed the first to our knowledge ddPCR protocol, which, in theory, can be used with any sample type (parasite eggs, larval cultures, grass samples, etc.) containing a mixture of DNA from both *Cooperia* sp*.* and *Ostertagia* sp. parasites, for their identification and absolute quantification.

In contrast to the previous work, which also utilized the ddPCR platform for the quantification of different genera of strongyle nematodes of sheep, based on uniplex reactions (Elmahalawy et al. 2018a), the present study takes a novel and unique approach, wherein both genera of parasites were amplified using the same primer pair, but distinguished and quantified using different probes in duplex reactions. This is an important advantage from an analytical point of view, especially when considering the varying efficacy of different PCR reactions, which may induce a small but systematic bias (Ruijter et al. 2015). In addition, although more challenging to develop, multiplex PCR assays are considered to be of higher utility, specifically for monitoring purposes (Hunt 2011).

On top of the abolishment of the need for standards, increased tolerance to PCR inhibitors, and the existing availability of fully automatized protocols, the precise and robust quantification of DNA sequences is one of the few areas where ddPCR truly excels in comparison with similar technologies, such as qPCR. Another is the capacity to run multiple probes per single reaction. Unlike the previous qPCR studies (Drag et al. 2016; Höglund et al. 2013), where only a single primer pair/probe set was utilized per one reaction volume, the ddPCR platform enables the use of two probes for competitive or non-competitive binding assays, thus presenting more alternative approaches to making a straightforward, fully automatized assay, which requires little time spent at the bench.

Herein, we have validated three different probes, all of which were designed to bind to the ITS2 region of *Cooperia* and/or *Ostertagia* DNA. The first, universal, probe was designed to indiscriminately anneal to the same region in both genera of parasites, whereas the remaining two specific probes to either of them (Fig. 1). Through in silico analysis of *Cooperia oncophora* and *Ostertagia ostertagi* ITS2 region sequences, retrieved from the NCBI database, we observed a single nucleotide difference in the consensus sequences between the parasite genera and decided to exploit this by manufacturing the two previously mentioned genus-specific probes. The sequence for the universal probe, although somewhat overlaps with the one describe by Elmahalawy et al. (2018a), was shifted upstream from the said single nucleotide difference, since the area surrounding it was found to be exceptionally homogenous.

Both LoD and FA precision tests produced promising results (Figs. 2 and 3). The LoD experiment provided robust FA values for the detection of each individual parasite genus while in the presence of the other (2.2% for *Ostertagia* and 0.67% for *Cooperia*). The FA precision test demonstrated an accurate identification of FA indices, displaying a steady and reproducible pattern, when the two parasite genera DNA samples were mixed together at different ratios. Furthermore, the obtained quantification results for the three different probes (Fig. 4) showed a rather precise agreement between the genus-specific probes and the universal one. It is certainly the case that random DNA copy variation upon serial dilutions and pipetting errors did account for some inaccuracies in the final measurements; however, this does not seem to diminish the significance of the quantification results obtained by the genus-specific and universal probes in all three previously discussed experiments. Taking probe cross-reactivity into account and through mere trial and error, we established firm manual thresholds for the acceptance of positive droplets in the case of both genus-specific probes: 8000 AU (Arbitrary units) for *Cooperia* (FAM™ fluorescent molecule) and 5000 AU for *Ostertagia* (HEX™ fluorescent molecule). A preliminary 8000 AU threshold was also set for the universal probe–generated positive droplets to limit the inclusion of false positives. However, the adjustment of the latter had virtually no impact on the outcome of the final quantification (the reduction of the threshold from 8000 to 4000 AU yielded the difference of, at most, 4 copies/μL). Even though in some *Cooperia-* and *Ostertagia*-only DNA samples vestigial amounts of the other parasite DNA was detected (in Fig. 4, “*Cooperia*” and “*Ostertagia*” samples), we disregarded them as the outcome of minimal probe cross-reactivity and have, thus, concluded that the probes are capable of effectively distinguishing sequences belonging to each parasite genus.

With the rather recent rise in the emergence of drug-resistant strains of cattle parasites, it is more important than ever to rigorously monitor for the presence, abundance, and proportion of each these nematodes at farms at risk. Here, we present a novel approach for molecular identification and precise quantification of the two major genera of parasitic GIN of cattle, i.e., *Cooperia* and *Ostertagia*, using the ddPCR platform on DNA samples derived from infected grazing cattle. Even though, further studies are required to adapt this technique to fecal and grass samples, the assay described in this paper is a first step and a necessary proof of the concept.

## Electronic supplementary material

Supplementary figure 1 1D plot displaying the ddPCR temperature gradient experiment, run on both parasite genera DNA (Blue droplets represent those containing *Cooperia* DNA, green – *Ostertagia* DNA) at different temperatures. ddPCR was run on *Cooperia* and *Ostertagia* DNA using the specific primer/probe pairs for each parasite genera at 8 different temperatures (an interval between 50 to 60 °C), as indicated above the positive droplet bands in both cases. 57 °C was further selected as the most optimal annealing temperature. A01-H01 and A02-H02 correspond to different wells, containing the generated droplets (along with either *Cooperia* or *Ostertagia* DNA), in a 96-well plate, while the Amplitude displays the intensity of fluorescence of each generated droplet (otherwise referred to as AU or Arbitrary units). Different thresholds at 8000 AU (for channel 1, detecting FAM produced fluorescence) and 5000 AU (for channel 2, detecting HEX produced fluorescence) can be seen as lines in blue and pink, respectively (PDF 6236 kb)

ESM 1 (PNG 169 kb)
