# Supplementary figures and images for: Molecular detection of two major gastrointestinal parasite genera in cattle using a novel droplet digital PCR approach
Source: Parasitol Res. 2019 Aug 6;118(10):2901–7. doi: 10.1007/s00436-019-06414-7 (PMC6754349; doi:10.1007/s00436-019-06414-7)

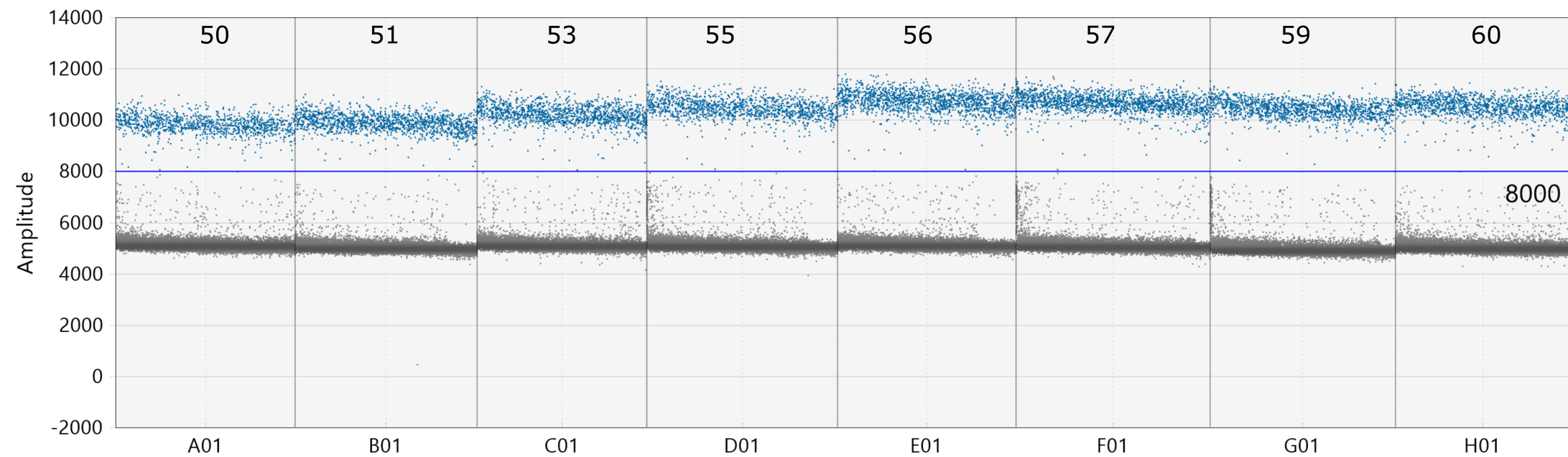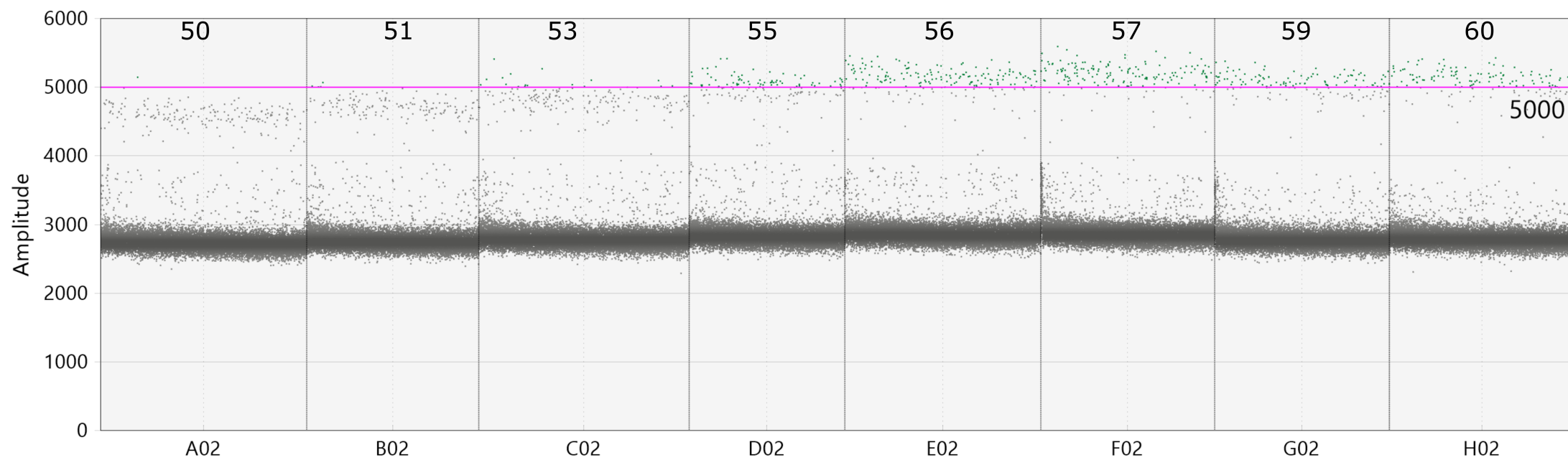

Supplement: Supplementary file 1 — 1D plot displaying the ddPCR temperature gradient experiment, run on both parasite genera DNA (Blue droplets represent those containing Cooperia DNA, green – Ostertagia DNA) at different temperatures. ddPCR was run on Cooperia and Ostertagia DNA using the specific primer/probe pairs for each parasite genera at 8 different temperatures (an interval between 50 to 60 °C), as indicated above the positive droplet bands in both cases. 57 °C was further selected as the most optimal annealing temperature. A01-H01 and A02-H02 correspond to different wells, containing the generated droplets (along with either Cooperia or Ostertagia DNA), in a 96-well plate, while the Amplitude displays the intensity of fluorescence of each generated droplet (otherwise referred to as AU or Arbitrary units). Different thresholds at 8000 AU (for channel 1, detecting FAM produced fluorescence) and 5000 AU (for channel 2, detecting HEX produced fluorescence) can be seen as lines in blue and pink, respectively (PDF 6236 kb) [file 436_2019_6414_MOESM1_ESM.pdf]

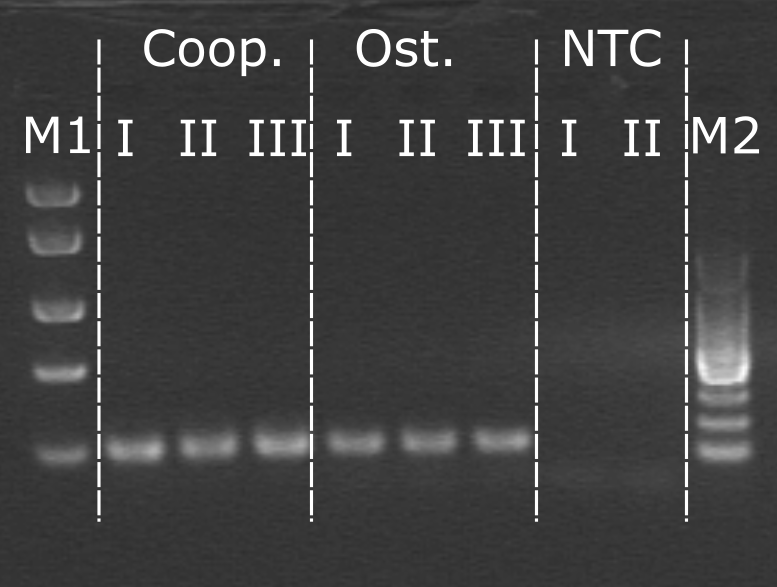

Supplement: Supplementary file 2 — (PNG 169 kb) [file 436_2019_6414_MOESM2_ESM.png]
